# Comparative Genome Analysis of *Bacillus amyloliquefaciens* Focusing on Phylogenomics, Functional Traits, and Prevalence of Antimicrobial and Virulence Genes

**DOI:** 10.3389/fgene.2021.724217

**Published:** 2021-09-30

**Authors:** Hualin Liu, Vimalkumar Prajapati, Shobha Prajapati, Harsh Bais, Jianguo Lu

**Affiliations:** ^1^School of Marine Sciences, Sun Yat-sen University, Zhuhai, China; ^2^Division of Microbiology and Environmental, Biotechnology, Aspee Shakilam Biotechnology Institute, Navsari Agricultural University, Surat, India; ^3^SVP-A School of Sardar Vallabhbhai National Institute of Technology, Surat, India; ^4^Delaware Biotechnology Institute, University of Delaware, Newark, DE, United States; ^5^Southern Marine Science and Engineering Guangdong Laboratory, Zhuhai, China

**Keywords:** *B. amyloliquefaciens*, phylogenomics, genome evaluation, comparative genomics, functional traits, antimicrobial resistance and virulence genes

## Abstract

*Bacillus amyloliquefaciens* is a gram-positive, nonpathogenic, endospore-forming, member of a group of free-living soil bacteria with a variety of traits including plant growth promotion, production of antifungal and antibacterial metabolites, and production of industrially important enzymes. We have attempted to reconstruct the biogeographical structure according to functional traits and the evolutionary lineage of *B. amyloliquefaciens* using comparative genomics analysis. All the available 96 genomes of *B. amyloliquefaciens* strains were curated from the NCBI genome database, having a variety of important functionalities in all sectors keeping a high focus on agricultural aspects. In-depth analysis was carried out to deduce the orthologous gene groups and whole-genome similarity. Pan genome analysis revealed that shell genes, soft core genes, core genes, and cloud genes comprise 17.09, 5.48, 8.96, and 68.47%, respectively, which demonstrates that genomes are very different in the gene content. It also indicates that the strains may have flexible environmental adaptability or versatile functions. Phylogenetic analysis showed that *B. amyloliquefaciens* is divided into two clades, and clade 2 is further dived into two different clusters. This reflects the difference in the sequence similarity and diversification that happened in the *B. amyloliquefaciens* genome. The majority of plant-associated strains of *B. amyloliquefaciens* were grouped in clade 2 (73 strains), while food-associated strains were in clade 1 (23 strains). Genome mining has been adopted to deduce antimicrobial resistance and virulence genes and their prevalence among all strains. The genes *tmrB* and *yuaB* codes for tunicamycin resistance protein and hydrophobic coat forming protein only exist in clade 2, while *clpP*, which codes for serine proteases, is only in clade 1. Genome plasticity of all strains of *B. amyloliquefaciens* reflects their adaption to different niches.

## Introduction

Since the 19th century, Bacilli is one of the most well-documented and preeminently characterized bacterial genera comprising classical microbiology, biochemistry, and advanced genomic and proteomic approaches (Alcaraz et al., 2010). Among the various species of bacilli, *Bacillus amyloliquefaciens* gains lots of research interest and has wide application in agriculture, pharmaceuticals, food industry, environmental industry, etc. (Sharma and Satyanarayana, 2013). Various strains of *B. amyloliquefaciens* are common habitants and frequently screened from various ecological niches, including soil, animal feces, human food, aquatic environments, and many more, reflecting its versatile metabolic capabilities (Earl et al., 2008). During evolution, the bacterial population acclimatized to their respective ecological niches, which lead to the differentiation as evidenced by various genomic and physiological characteristics (De Wit et al., 2012).

Versatility of nature and metabolic competencies of different strains of *B. amyloliquefaciens* provoke to expedite the comparative genomic analysis to address more in detail the life style of bacteria, their adaptation to various niches and how they overcome contenders, as well as to catch clear revelation on their biochemistry, physiology, and genetics (Sharma and Satyanarayana, 2013; Owusu-Darko et al., 2020). *B. amyloliquefaciens* have been known to promote plant growth via a variety of mechanisms (Baghaee Ravari and Heidarzadeh, 2014; Shao et al., 2015; Liu et al., 2016), act as biocontrol against numerous plant diseases caused by soil-borne microorganisms (Tan et al., 2016), be widely used as biofertilizers and biopesticides (Wu et al., 2015), antagonize plant pathogens by competing essential nutrient (Wu et al., 2016), produce antibiotic compounds (Srivastava et al., 2016), as well induce systemic acquired resistance (Ng et al., 2016). Moreover, it is well documented that *B. amyloliquefaciens* can be tailored for numerous environmental and industrial applications such as degradation of crude oil from oil-contaminated sites (Zhang J. et al., 2016) and feather degradation (Yang et al., 2016); can produce various enzymes like proteases (Wang et al., 2016), feruloyl esterase (Wang et al., 2017), phytase (Verma et al., 2016), and amylases (Prajapati et al., 2015); and can be employed as a biosorbent for the removal of pollutants (Sun et al., 2016) and their degradation (Zühlke et al., 2016), production of biosurfactant and AMPs, probiotics, etc. (Perez et al., 2017).

The number of bacterial genome sequences has almost doubled over the decades due to the decreasing cost of the sequencing with advancement in high-throughput sequencing technology. The generated sequences data are available freely in the public domain, which ultimately stimulate researchers to do more on genomic analysis. Comparative genome analysis always sharpens our understanding of the bacterial genome structure and its diversity at a particular niche. Moreover, the pan-genome of species includes analysis of all core genes, dispensable genes, and strain-specific genes, which need to be comprehensively investigated as they reveal the essential functions for the species or laterally transferred functions in specific strains (Vernikos et al., 2015). *Bacillus* is one of the most extensively studied species with prevalent sets of genome sequences to date; however, very few reports are available on core genes and strain-specific genes in the *Bacillus* species (Alcaraz et al., 2010). Kim et al. (2017) have reported the core gene data of multiple *Bacillus* species through pan-genome analysis to explore the *Bacillus* species in food microbiome.

In the present investigation, we have curated all the 96 genome sequences of *B. amyloliquefaciens* available in the NCBI database to carry out comparative genomic analysis. Based on contextual information, we were trying to understand the distribution of all strains of *B. amyloliquefaciens* with respect to their ecological niches and their source of isolation and location to get better insights into their phylogenetic position using the core genome. PAN genome analysis of all strains of *B. amyloliquefaciens* was conducted to acquire better impression on their functional difference, which affects their dynamic evolutionary processes. We were also interested in understanding the comparative account of various antimicrobial and virulence genes presented among all *B. amyloliquefaciens* strains. The consensus information and conclusion drawn from this presented comparative genomic study can be used as a benchmark for designing wet-lab experimentation and validation as well as to formulate new hypothesis.

## Materials and Methods

In total, 96 genome sequences of *B. amyloliquefaciens* having an N50 size greater than 50 k were downloaded from the NCBI database (detailed in Supplementary Table 1). Pan-genome analysis was conducted by Roary (Page et al., 2015) embedded in the “Pan” module of PGCGAP v1.0.21 (Liu et al., 2020). Single-copy core proteins calling, alignment of sequences, sequences concatenating, best model chosen, and phylogenetic tree constructing were performed with the “CoreTree” module of PGCGAP v1.0.21. The pairwise similarity of genomes was calculated by Mash (Ondov et al., 2016) embedded in the module “MASH” of PGCGAP v1.0.21. COG annotation was performed with the module “pCOG” of PGCGAP v1.0.21 (Liu et al., 2020). The antimicrobial resistance and virulence genes were mined against the databases of argannot (Gupta et al., 2014), card (Jia et al., 2017), NCBI (Feldgarden et al., 2019), resfinder (Zankari et al., 2012), vfdb (Chen et al., 2016), and EcOH (Ingle et al., 2016) by the module “AntiRes” of PGCGAP v1.0.21 (Liu et al., 2020).

## Results

**FIGURE 1:**
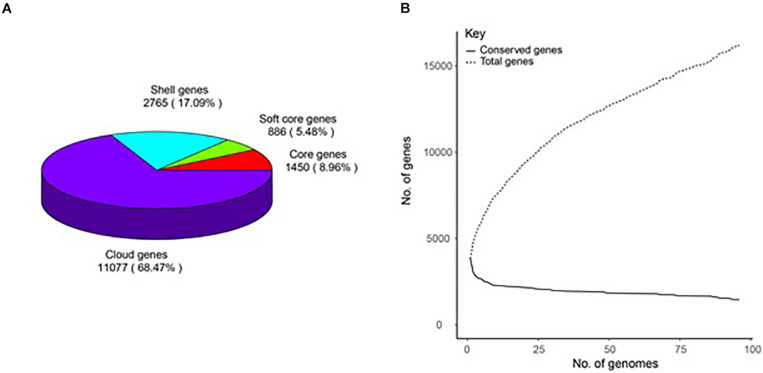
Pan-genome of 96 *B. amyloliquefaciens*. **(A)** A pie chart of the breakdown of genes (core: 99% ≤ strains ≤ 100%, soft-core: 95% ≤ strains < 99%, shell: 15% ≤ strains < 95%, and cloud: 0% ≤ strains < 15%). **(B)** The pan-genome curve of the 96 genomes.

A total of 16,198 gene clusters were found by pan-genome analysis, of which 1,448 (8.95%) are single-copy and code for core proteins. Shell genes, soft-core genes, core genes, and cloud genes comprise 17.09, 5.48, 8.96, and 68.47%, respectively, which demonstrates that the genomes are very different in the gene content (Supplementary Table 2). The pan-genome curve shows that the number of total genes increased with the increase in the genome number; this indicates that *B. amyloliquefaciens* has an open pan-genome (Figure 1).

**FIGURE 2:**
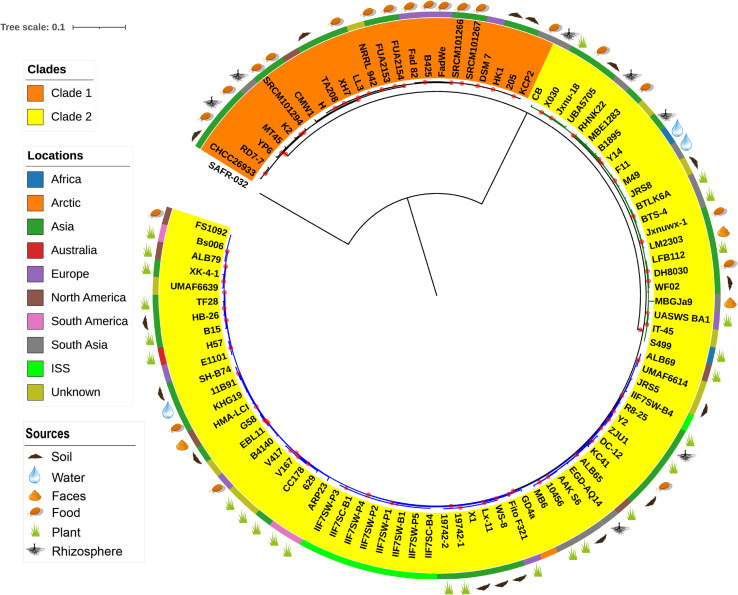
The phylogenetic tree of single-copy core proteins. The maximum-likelihood tree was constructed by the module “CoreTree” of PGCGAP v1.0.21 (Liu et al., 2020) with the best fit model JTT+F+R4. The tree was rooted against *Bacillus pumilus* SAFR-032 as an appropriate outgroup. Red circles on the branch represent bootstrap values larger than 80%. The background color of the labels (colored ranges) represents the clades, and the green and blue branches represent cluster 1 and cluster 2 of clade 2, respectively. The color strip outside the tree describes where the strain was isolated; ISS means International Space Station. The cartoons outside the tree indicate that the strain was isolated from soil, food, rhizosphere, water, or faces of herbivores, or is associated with the plant.

The evolutionary relationship between the 96 *B. amyloliquefaciens* strains was investigated by the construction of a phylogenetic tree based on the alignment sequences of 1,154 concatenation core proteins (Figure 2). *Bacillus pumilus* SAFR-032 (Gioia et al., 2007) was used as the outgroup. The strains are divided into two clades, and clade 2 consists of two clusters. The location where the strain was isolated was mapped outside of the tree as the color strip. Strains from America are mainly located in cluster 2 of clade 2, while strains from Asia and Europe are scattered in all clades. The isolation source of the strain was also marked on the tree. According to known information, almost all the plant-associated strains are located in clade 2, and strains isolated from food are mainly located in clade 1. The above result implies that *B. amyloliquefaciens* has differentiated mainly into plant-associated and food-associated, as it clearly showed in the clades. However, some species of *B. amyloliquefaciens* isolated from water, soil, etc. are scattered in clade 2.

**FIGURE 3:**
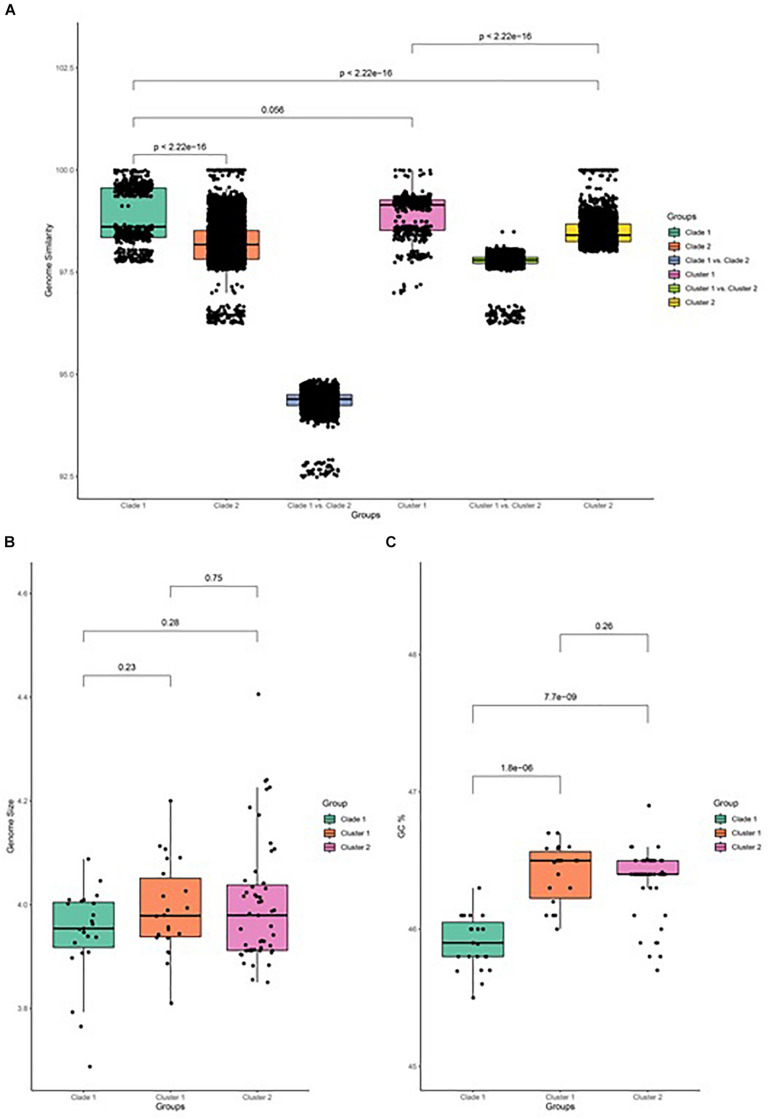
Genome feature of *B. amyloliquefaciens*. **(A)** Genome similarity between all pairs of strains in clade 1, between all pairs of strains in clade 2, between strains in clade 1 and those in clade 2, between strains in cluster 1, between strains in cluster 2, and between strains in cluster 1 and those in cluster 2. **(B)** Genome size of clade 1 and cluster 1 and cluster 2 of clade 2. Wilcox test was performed and marked on top of the box plot. **(C)** GC percent of clade 1, cluster 1, and cluster 2 of clade 2. Wilcox test was performed and the *p*-value was marked on top of the box plot.

The similarity of genome pairs has been compared within and between clades and clusters (Figure 3). Genomes in clade 1 are found to be more similar than those that are observed in clade 2 (*p* < 0.001), while the similarity between genomes of the two clades is found to be very low, which indicates that strains in clade 2 undergone more differentiation, which may be related to their adaption to specific plants and other associated niches. When focusing on clade 2, genomes in cluster 1 are more similar than genomes in cluster 2 (*p* < 0.001), and the genome similarity between the two clusters is seen to be relatively low. Comparison of the genome size between both clades and its associated cluster has been carried out and depicted in Figure 3B. It has been observed that the genome size of clade 2 is slightly greater than that of clade 1, while the GC% content of clade 2 is significantly greater than that of clade 1 (*p* < 0.001; Figure 3C).

**FIGURE 4B:**
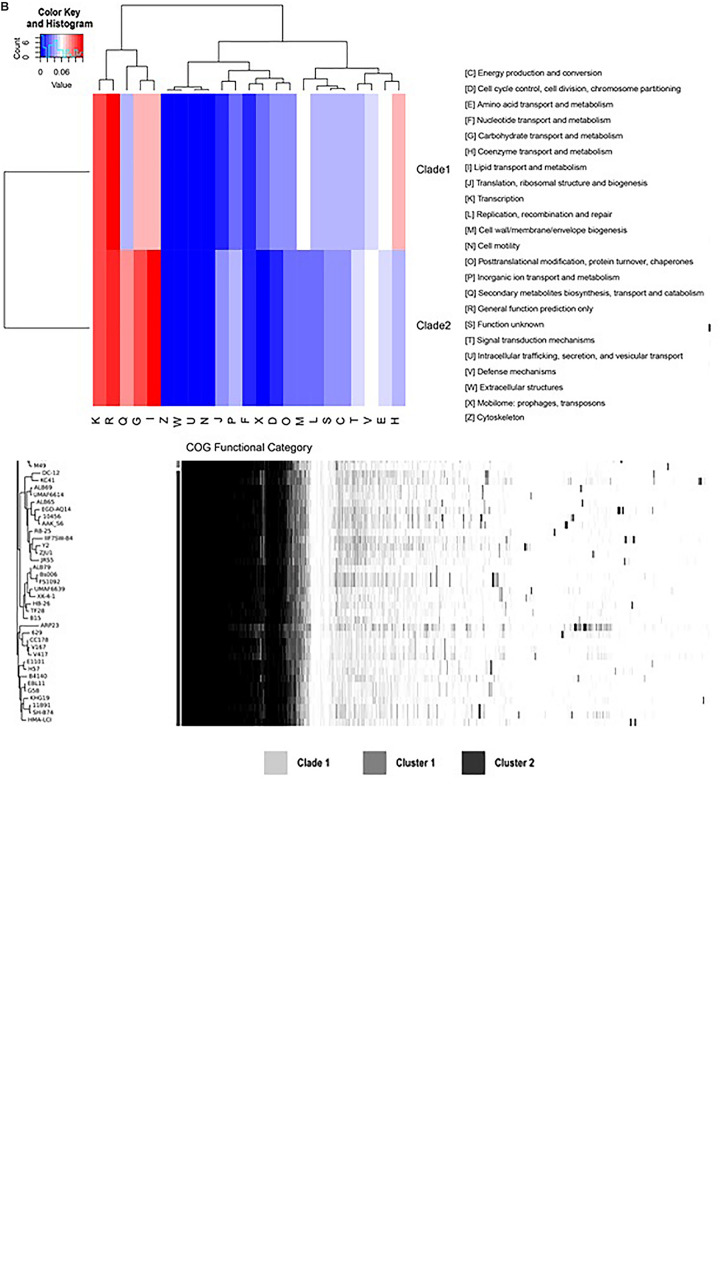
Comparison of gene families between clade 1 and clade 2. The plot shows the tree compared to a matrix with the presence and absence of core and accessory genes **(A)**; the bars between the tree and the matrix show to which clade/cluster the strain belongs. The heat map shows the relative abundance of the function classes corresponding to the unique orthogroups of clade 1 and clade 2 **(B)**.

Compared with the genomes of clade 2, the genomes of clade 1 have a unique gene composition (Figure 4A). It was observed that all the species in clade I have lost 335 genes (Supplementary Table 2 lines 2,592–2,926), which exists in all the genomes of clade 2 and have 490 unique core genes (Supplementary Table 2 lines 3,969–4,458). To reveal the difference of gene contents between the two clades, the gene family analysis has been performed with module “OrthoF” of PGCGAP v1.0.21. A total of 9,245 orthogroups are found, out of which 4,872 orthogroups are observed to be common between the two clades, while 1,055 are unique to clade 1, and the remaining 3,363 are unique to clade 2. The functions of these unique orthogroups are revealed through COG annotation as shown in Figure 4B. The relative abundance of functional classes I (lipid transport and metabolism), G (carbohydrate transport and metabolism), and Q (secondary metabolites biosynthesis, transport, and catabolism) is found to be higher in clade 2 compared to that in clade 1, while the relative abundance of classes D (cell cycle control, cell division, chromosome partitioning), E (amino acid transport and metabolism), H (coenzyme transport and metabolism), L (replication, recombination, and repair), M (cell wall/membrane/envelope biogenesis), and X (Mobilome: prophages, transposons) is higher in clade 1 than that in clade 2 (Figure 4B).

**FIGURE 5:**
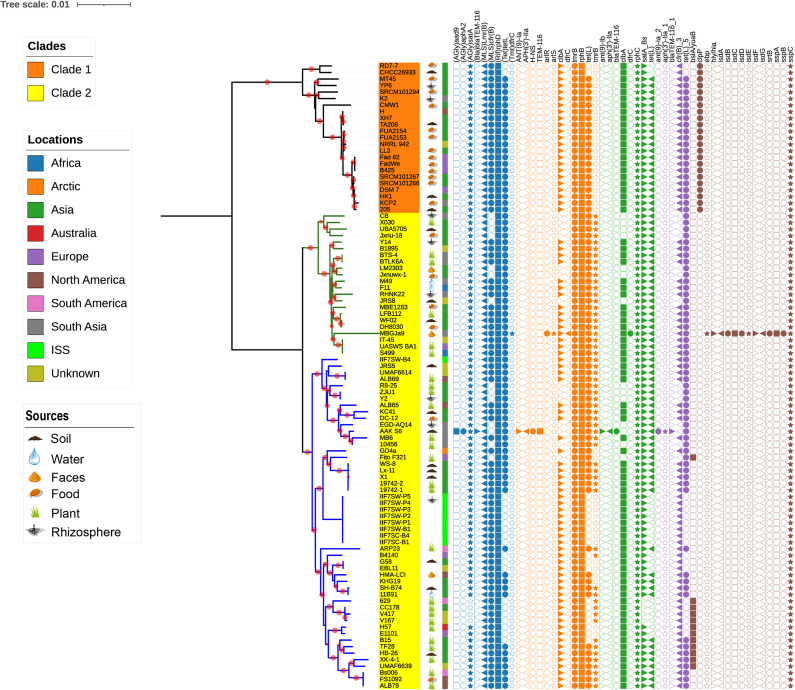
The midpoint rooted phylogenetic tree of single-copy core proteins. The tree was constructed by the module “CoreTree” of PGCGAP v1.0.21 (Liu et al., 2020) with the best fit model JTT+F+R4. Red circles on the branch represent bootstrap values larger than 80%. The background color of the labels represents the clades, and the green and blue branches represent cluster 1 and cluster 2 of clade 2, respectively. The cartoons after the strain name indicate that the strain was isolated from soil, food, rhizosphere, water, or faces of herbivores, or is associated with the plant. The color strip outside the tree describes where the strain was isolated; ISS means International Space Station. The symbols on the right side of the tree represent antibiotic resistance genes or virulence genes from each database [blue: argannot (Gupta et al., 2014), orange: card (Jia et al., 2017), green: NCBI (Michael Feldgarden et al., 2019), purple: resfinder (Zankari et al., 2012), and gray: vfdb (Chen et al., 2016)].

It is well documented that antimicrobial resistance and virulence genes are disseminated in the environment according to the function of the respective ecological niche; therefore, we have investigated the distribution of these genes in *B. amyloliquefaciens*. The antimicrobial resistance and virulence genes from different databases have been mined and mapped on the phylogenetic tree (Figure 5). To demonstrate the topological structure of the tree more clearly, the outgroup strain has been removed and the tree presented on midpoint rooted. All strains of *B. amyloliquefaciens* including those from foods contain more than one virulence factor. It is observed that *tmrB* and *yuaB* are only existing in clade 2, while *clpP* is prevailing only in clade 1. The gene *tmrB* is intending an ATP-binding tunicamycin resistance protein found in *B. subtilis* (Noda et al., 1995), while *yuaB* can form a highly hydrophobic coat around *B. subtilis* biofilms (Kobayashi and Iwano, 2012). The gene *clpP* codes for a serine protease involved in proteolysis and is required for growth under stress conditions (Gaillot et al., 2000, 2001). Interestingly, the *B. amyloliquefaciens* strain MBGJa9 has more virulence factors than other strains, and it is seen that *isdA*, *isdB*, *isdC*, *isdD*, *isdE*, *isdF*, *isdG*, and *srtB* form a gene cluster, whose productions participated in the uptake of iron and heme (Skaar and Schneewind, 2004; Skaar et al., 2004).

## Discussion

### Pan-Genome Assessment of *Bacillus amyloliquefaciens*

Present investigation using the 96 strains of *B. amyloliquefaciens* revealed that it has an extensive pan-genome, and it represents an ample number of genes that were observed to be uniquely associated with each of the divergent species. Population size and respective ecological niche versatility of *B. amyloliquefaciens* are considered to be the most influential factors in determining the pan-genome size, and it can be seen that total genes against the total number of genome sequences are edified up so it is impossible to envisage the size of the full pan-genome. The resulted open pan-genome of *B. amyloliquefaciens* is unsurprising because of the source of isolation and its geographical location, which always adds up new genes to the entire gene pool of species. This species divergence could be an attribute of different mechanisms such as horizontal transfer, transposition, and transformation (Konstantinidis and Tiedje, 2005; Tettelin et al., 2005). On the contrary, observed few core genes in the investigation might be due to the higher number of genomes, the incorporation of genomes from other genera, as well as the inclusion of draft genomes in the data set (Lefebure et al., 2010; Inglin et al., 2018). It is well documented that incomplete, unfinished, or partially assembled genomes have a large impact on the occurrence of core genomes in the analysis as core genomes seem to be very sensitive to the heterogenous data set and poor quality sequences (Mendes-Soares et al., 2014).

### The Alliance of *B. amyloliquefaciens* Strains Through Phylogenomics Using Single-Copy Core Proteins and Genomic Comparison: An Evolutionary Assessment

16s rRNA has been widely used for the taxonomy assessment of prokaryotes and has served as the broad context, though the better taxonomic resolution of the microbial species is achieved through “polyphasic approach” and is highly effective (Rosselló-Mora and Amann, 2001; Na et al., 2018). 16s rRNA has limitations as it hampers the phylogenic resolution at the species or subspecies level. The application of genome sequences is highly recommended for the taxonomic understanding of microbial species instead of routinely used DNA–DNA hybridization and 16s rRNA phylogeny (Chun et al., 2018). Therefore, instead of a single gene, genome-based phylogeny called phylogenomics has set up better taxonomic positioning as it uses sets of core genes (Eisen and Fraser, 2003). The genome sequences of all *B. amyloliquefaciens* strains are accessible in the Gene Bank NCBI database, which allows us to determine the degree of genome variability among all species as well as distinct out the taxonomic validity of all the isolates and reconstruct their phylogenetic relationship. Two distinct clades were observed when phylogeny was inferred using single copy core protein. Clade 1 comprises 23 strains of *B. amyloliquefaciens*, out of which 56% were food-associated, 17.39% were from soil, and 8.69% were rhizospheric. Clade 2 comprises 73 strains, and it is distinguished into two different clusters, where clusters 1 and 2 comprise 22 and 51 strains of *B. amyloliquefaciens*, respectively. Clade 2 was more enriched with the species of plant origin/host and comprised ∼35.61%, while the strains of soil, food, indoor biome, and rhizosphere origin were 16.43, 10.95, 12.32, and 6.84%, respectively. Two distinct clades were demarcated, one of which was food-associated (clade 1) and the other one plant-associated (clade 2). The selection of core gene sets for accurate phylogeny analysis may vary with the availability of the genome sequences at the time of analysis.

Comparison of the genome similarity between the strains of both clades indicates that the strains grouped together in clade 1 are more similar than those of clade 2. The majority of the plant-associated strains of *B. amyloliquefaciens* are grouped under clade 2, while nonplant-associated strains are mainly found in clade 1, though some scattering is seen with respect to some other ecological niches. Plant-associated strains of *B. amyloliquefaciens* have adopted more modification in their genome, which is directly related to their adaption to the specific plant. Hence, it is believed that the genome size of the plant-associated strains of *B. amyloliquefaciens* is always greater than that of the nonplant-associated *B. amyloliquefaciens* and so the GC % content. Zhang N. et al. (2016) reported that the core genomes of the plant-associated strains of *B. amyloliquefaciens* have more gene contents related to the intermediary metabolism and secondary metabolite biosynthesis as compared to those of nonplant-associated strains. Plant-associated strains also possess specific genes for the synthesis of antibiotics as well as for the utilization of plant-derived substrates.

During the assessment of the core and accessory genes, it was observed that the strains of *B. amyloliquefaciens* grouped in clade 1 have lost many genes that are present in the strains of clade 2 (Figure 4A). Exopolysaccharides (EPSs) play very important role in bacteria, specifically those that are plant-associated and have a variety of functions. It helps microorganisms in adherence, pathogenesis, and symbiosis as well as protects from desiccation in some adverse condition (Stingele et al., 1999). The glycosyltransferase gene region comprises the EPS gene cluster, i.e., *epsF-2, epsD, epsI, epsM, epsL*, and *epsJ*, which are involved in the biosynthesis of EPS, and has a profound role in plant-associated strains of *B. amyloliquefaciens*, while it was missing in the strains belonging to clade 1. Plant-associated *B. amyloliquefaciens* strains (clade 2) harbor a certain gene cluster absent in clade 1, which is involved in the biosynthesis of lipopeptides through nonribosomal peptide synthetases (NRPS) including fengycin (*fen*). Gene clusters involved in the synthesis of bacillaene (*bae*) are responsible for the profound antimicrobial activity and are lost in all strains of *B. amyloliquefaciens* in clade 1. The PKS gene cluster, which includes *pksI_2, pksG_2, pksN_2*, and *pksS*, was also found to be present in clade 2 but lost in clade 1 (Figure 4A). Some of the genes such as cystathionine beta-lyase (*patB*), putative multidrug resistance ABC transporter ATP-binding/permease protein (*yheI*), cold shock protein (*cspC*), spermidine/spermine N(1)-acetyltransferase (*paiA*), putative sugar phosphate isomerase (*ywlF*), 3-dehydroshikimate dehydratase (asbF), putative ABC transporter substrate-binding lipoprotein (yhfQ), sirohydrochlorin ferrochelatase (sirB), putative metallo-hydrolase (yflN), dipeptidyl-peptidase 5 (ddp5), L-aspartate oxidase (nadB), putative sporulation hydrolase (cotR), stress response kinase A (srkA), sortase D (srtD), ATP-dependent dethiobiotin synthetase (bioD 1), glycerophosphodiester phosphodiesterase (glypQ), folylpolyglutamate synthase (fpgS), and putative ABC transporter permease (ytrC) were found to be uniquely associated to the strain of *B. amyloliquefaciens* that belongs to clade 1. Hence, the presence of certain gene clusters in clade 2 and their absence in clade 1 conclude that plant-associated strains of *B. amyloliquefaciens* have more abundant gene clusters for intermediary metabolism as well as for antibiotic production compared to the nonplant-associated strains. Niazi et al. (2014) have reported that *B. amyloliquefaciens* subsp. *plantarum*, a rhizobacterium that mends plant growth and stress management, also possesses the more abundant gene cluster that is actively involved in the production of certain hydrolytic enzymes as well as secondary metabolites. It is well documented that the rhizosphere environment has a very dynamic microbial community because of the effect of root exudates and the constant interaction and competition among microbes, as they need to contend with each other for various resources such as nutrient supply, which ultimately leads them to produce various metabolites such as antibiotic and extracellular hydrolases (Bais et al., 2006).

### Surveillance of Resistance and Virulence Genes Among all Strains of *B. amyloliquefaciens*

It is documented that bacteria have produced antibiotics for millions of years, which results in the evolution and induction of resistance genes. More precisely, the intensive nonmedical use of antibiotics such as in agricultural and in some industrial applications is not certain and has led to significant dissemination of resistance genes in the environment (Pawlowski et al., 2018). The genomes of all the strains of *B. amyloliquefaciens* were mapped to different databases to evaluate the distribution of antibiotic resistance genes and virulence genes. Many different genes were perceived and were scattered among all the strains of *B. amyloliquefaciens*; also, the observed genes belonged to a variety of resistance classes. The gene (AGlu) *satA* codes for the enzyme aminoglycoside acetyltransferase, and it belongs to the class aminoglycosidese, which is present in almost all the strains independent of its host environment. Aminoglycoside is considered to be part of the broad spectrum of antibiotics, and it acts by inhibiting the protein synthesis, though it works best in synergy with other antimicrobials (Krause et al., 2016). Two genes, *lmrB* and *cfrB*, belonging to the class Macrolide-Lincosamide-Streptogramin B (MLS) were present in most of the strains considered in the investigation. The gene product of *lmrB* and *cfrB* confers specific resistance to lincosamides, such as lincomycin and clindamycin, and synthetic antibiotic linezolid, respectively, (Kim et al., 2001; Toh et al., 2007). The advent of new and more stable macrolide and its vague use could be the key reasons for the induction of such resistance imparting genes, and it provides an opportunity for microbial populations to acquire MLS resistance (Roberts et al., 1999). (Rif) *rphD, rphB*, and *rphC* genes code for trifamycin kinase (phosphotransferase), which confers resistance against rifampin, the most commonly used rifamycin. The enzyme rifampin phosphotransferase present in many environmental bacteria, which used to be induced by selective pressure and nonclinical use of antibiotics, has led to the inactivation of rifampin and ATP to phosphor-rifampin and AMP+Pi (Stogios et al., 2016). More than 40 different tetracycline resistance genes have been reported in numerous bacterial genera of agricultural and industrial use. The dispersion of the *tetL* gene among the bacterial genera was much higher than any other *tet* resistant genes (Roberts, 2005). In the present investigation, the genome sequences of all the strains were mapped against six different databases, i.e., argannot, NCBI, plasmidfinder, card, resfinder, and yfdb, and they reveal the presence of *tetL* genes among all the strains. Colibactin is a genotoxic molecule coded by the *clb* gene cluster in many enteric bacteria, and it is widely distributed in nature (Kawanishi et al., 2020). *clbA* is a plasmid-encoded cfr gene under the control of an inducible promoter reported in *B. velezensis* (*B. amyloliquefaciens* subsp. *plantarum*), while *clbB* and *clbC* are found in *Brevibacillus brevis* and *B. clausii*, respectively, (Hansen et al., 2012). The gene *sspC* codes for cytoplasmic protein known as staphostatin and is present in all the strains of *B. amyloliquefaciens*. It is a very specific and tightly binding inhibitor of staphopain B (SspB). The main function of *sspC* is to protect the cytosolic protein from the degradation executed by misfolded or activated SspB. Shaw et al. (2005) reported that in the absence of sspC protein, major alteration in cellular physiology occurred, and the growth and viability of the microbial cells were impaired. The gene *clpP* is prevailing only in the strains that belong to clade 1, and it codes for the caseinolytic protease proteolytic subunit (ClpP) serine proteases. The ClpP protein confers certain advantages to the microorganisms to sustain in varying environmental conditions as well as stress conditions. ClpC and ClpP are heat shock proteins and are subunits of ATP-dependent proteases reported in *B. subtilis*. The transcription of genes *clpC and* clpP is always negatively regulated under nonstressed condition (Krüger et al., 2001). The virulence and infectivity of a number of microorganisms/pathogens are affected due to the alteration of the ClpP protein function. Clp proteins are highly conserved and have played a very important role in the proteolysis of prokaryotic cell and eukaryotic organelles, though only few reports are available describing the importance of Clp-mediated proteolysis in organisms (Krüger et al., 2001; Moreno-Cinos et al., 2019). Tunicamycin, a nucleoside antibiotic, kills most of the gram-positive bacteria, and it acts by inhibiting the important cell wall component called teichoic acid, which drives the physiology and pathogenesis of microorganisms. The exposure of bacteria toward the sub-inhibitory concentration of tunicamycin leads to the reduction in biofilm production, virulence protein, as well bacterial adhesion and invasion (Zhu et al., 2018). The presence of the *tmrB* gene leads to the production of the TmrB protein, which imparts tunicamycin resistance to *B. subtilis*. The TmrB protein is present in both cytoplasmic and membrane fractions, though it is completely hydrophilic, and it attaches to the membrane by its C-terminal amphiphilic alpha-helix (Noda et al., 1995). Many plant growth promoting bacteria reported to produce biofilm, which is their key strategy to survive successfully in some harsh conditions as well as in plant rhizosphere. Biofilm formation capability of microorganisms makes them a good biocontrol agent as it leads to the reduction in infection caused by fungal and bacterial pathogens (Hobley et al., 2013). The *bslA/yuaB* gene present in many of the plant-associated strains of *B. amyloliquefaciens* codes for unique surface active protein BslA, which forms a hydrophobic surface layer called hydrophobins. The surface layer regulates the diffusion of various molecules, perception of signaling molecules from other microbial community, as well as nutrient uptake, in addition to imparting the protection to the bacterial cell. The contextual information of ecological and evolutionary facts as well as the application of comparative genomics and the dropping cost of genome sequencing collectively aid to understanding more precisely the structure of microbial diversity and its ecological distribution. Phylogenomics reveals the segregation of all 96 strains of *B. amyloliquefaciens* into two clades. Majority of the plant-associated *B. amyloliquefaciens* strains are grouped in clade 2, while clade 1 accomplishes mostly food-associated strains. The distribution of resistance and virulence genes among all the strains of *B. amyloliquefaciens* has been reported, and it will serve as a benchmark and resourceful information to deduce the hypothesis or conclusion as well as to exploit the potential of any strains through wet-lab experimentation. In future prospectus, we will try to dig out some temporal genes and their occurrence pattern in order to comprehend the significant role of microorganisms as well as the structure of the entire microbial community with its respective environmental niches.

## Data Availability Statement

The original contributions presented in the study are included in the article/Supplementary Material; further inquiries can be directed to the corresponding author/s.

## Author Contributions

VP conceived and modeled the study. VP and HL analyzed the data and prepared the methods and results. VP and SP prepared the manuscript. HB and JL corrected the manuscript and inputs. All authors contributed to the article and approved the submitted version.

## Conflict of Interest

The authors declare that the research was conducted in the absence of any commercial or financial relationships that could be construed as a potential conflict of interest.

## Publisher’s Note

All claims expressed in this article are solely those of the authors and do not necessarily represent those of their affiliated organizations, or those of the publisher, the editors and the reviewers. Any product that may be evaluated in this article, or claim that may be made by its manufacturer, is not guaranteed or endorsed by the publisher.

## Supplementary Material

Supplementary Figure 1 A flow chart representation of analyses conducted by different modules of PGCGAP v1.0.21, which integrates some popular software and in-house scripts (Liu et al., 2020).

Supplementary Table 1 Details of 96 *B. amyloliquefaciens* strains, including their genome size, GC%, scaffolds, CDS, its host and geographical location of the isolates, etc., retrieved from the NCBI database for comparative genome analysis.

Supplementary Table 2 Data analysis of pan-genome segregation to identify single-copy core proteins, shell genes, soft-core genes, core genes, and cloud genes, including its annotation, genome fragment and order within fragments, accessory order, and accessory order with fragment.
