# Supplementary figures and images for: Comparative Genome Analysis of Bacillus amyloliquefaciens Focusing on Phylogenomics, Functional Traits, and Prevalence of Antimicrobial and Virulence Genes
Source: Front Genet. 2021 Sep 30;12:724217. doi: 10.3389/fgene.2021.724217 (PMC8514880; doi:10.3389/fgene.2021.724217)

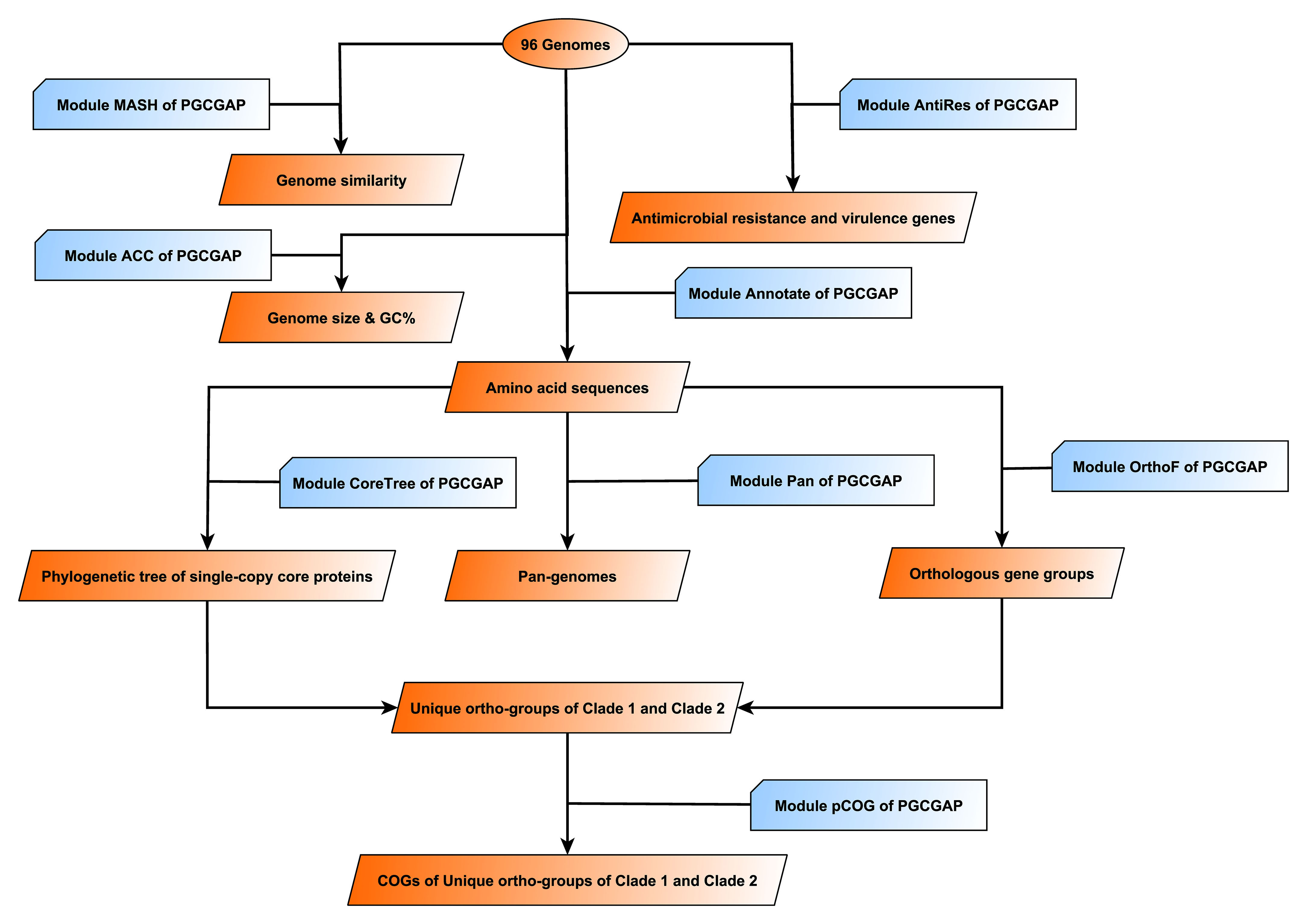

Supplement: Supplementary Figure 1 — A flow chart representation of analyses conducted by different modules of PGCGAP v1.0.21, which integrates some popular software and in-house scripts (Liu et al., 2020). [file Image_1.JPEG]
